# Clinical Significance of Screening Electrocardiograms for the Administration of Propranolol for Problematic Infantile Hemangiomas

**DOI:** 10.1155/2021/6657796

**Published:** 2021-02-23

**Authors:** James D. Phillips, Tyler Merrill, J. Reed Gardner, R. Thomas Collins, Jenika Sanchez, Adam B. Johnson, Brian K. Eble, Larry D. Hartzell, Jay M. Kincannon, Gresham T. Richter

**Affiliations:** ^1^Vanderbilt University Medical Center, Department of Otolaryngology Head and Neck Surgery, 1215 21st Ave S, Nashville, TN 37232, USA; ^2^University of Arkansas of the Medical Sciences, Department of Otolaryngology-Head and Neck Surgery, 4301 W Markham St Little Rock, AR 72205, USA; ^3^Stanford Children's Health, Department of Pediatrics, Cardiology Division, 725 Welch Rd. #120 Palo Alto, CA 94304, USA; ^4^University of Arkansas for the Medical Sciences, Department of Pediatrics, Cardiology Division, 1 Children's Way Little Rock, AR 72205, USA; ^5^University of Arkansas for the Medical Sciences, Department of Dermatology, 4301 W Markham Little Rock, AR 72205, USA

## Abstract

**Objective:**

Low-dose nonselective *β* blockade is an effective treatment for problematic infantile hemangioma (PIH). Screening electrocardiograms (ECG) are performed prior to the initiation of propranolol to minimize the risk of exacerbating undiagnosed heart block. How ECG results affect subsequent propranolol usage and patient management remains unclear. We examined the value of ECG prior to propranolol therapy in a quaternary pediatric hospital.

**Methods:**

A retrospective chart review was performed on all infants who received propranolol (2 mg/kg/day divided three times daily) to treat PIH at Arkansas Children's Hospital from Sept. 2008 to Sept. 2015. All available demographic, historical, and clinical data were obtained. ECGs and echocardiographic data were reviewed and summarized. A pediatric cardiologist read all ECGs.

**Results:**

A total of 333 patients (75% female) received propranolol therapy. ECG information was available for 317 (95%). Abnormal findings were present on 44/317 (13.9%) of study ECGs. The most common abnormal finding was “voltage criteria for ventricular hypertrophy” (*n* = 35, 76.1%). Two patients had abnormal rhythms; one had first-degree atrioventricular (AV) block, and one had occasional premature atrial contractions. Of the 31 patients who underwent echocardiograms, 20 (35%) were abnormal. 2.9% of infants with PIH treated with propranolol required a follow-up with a cardiologist. No patient was precluded from taking propranolol due to the findings on screening ECG.

**Conclusions:**

Screening ECGs prior to propranolol therapy are abnormal in nearly 14% of patients with PIH but are unlikely to preclude therapy. In the absence of prior cardiac history, this cohort offers further evidence suggesting that screening ECGs may be of limited value in determining the safety of propranolol in otherwise healthy infants with PIH.

## 1. Background

Infantile hemangioma is the most common vascular anomaly in children, affecting as many as 5-10% of infants in the first year of life [1–4]. Although the natural course of infantile hemangioma is self-limited, 10-15% of cases will eventually receive treatment [5]. Ulceration, bleeding, disfigurement, visual obstruction, airway compromise, high-output cardiac failure, and risk of persistent residuum are likely reasons for intervention [6].

After the serendipitous discovery of the efficacy of low-dose nonselective *β* blockade for problematic infantile hemangioma (PIH), oral propranolol has become the first-line intervention [7, 8]. Since its introduction, concern was raised that propranolol might expose otherwise healthy PIH patients to unintended cardiovascular effects. As a result, many vascular anomaly centers routinely admitted patients for cardiac observation and laboratory evaluation during the initiation of nonselective *β* blocker therapy [9]. As evidence of clinical safety increased, a multidisciplinary consensus guideline for pretreatment screening was released. This guideline included the recommendation that electrocardiographic evaluation be performed if a patient's history and physical examination revealed a lower than normal resting heart rate, arrhythmia, family history of arrhythmia, or maternal connective tissue disease in order to detect and prevent the exacerbation of an undiagnosed heart block [8]. Despite the robust use of ECG in screening PIH patients before the use of nonselective *β* blockage, its utility remains unclear. In addition, the impact of electrocardiograms (ECGs) on propranolol usage in PIH and the management of PIH patients with abnormal findings are uncertain. Recent articles have thereby questioned its pretreatment value [10]. Even extended monitoring, through the use of Holter monitors, has shown no contraindications to propranolol therapy despite significant changes in hemodynamics [11]. Frongia et al. indicated that despite the discovery of pathologies with pretreatment monitoring, in the form of ECG and echocardiograms, no contraindications were discovered [12]. With these concepts in mind, the present study examined the use and impact of ECG assessment prior to propranolol therapy in a high-volume vascular anomaly quaternary pediatric hospital.

## 2. Methods

After approval by the University of Arkansas for Medical Sciences Institutional Review Board (IRB# 202187), a retrospective chart review was performed on all infants who were offered propranolol therapy for PIHs at Arkansas Children's Hospital from Sept. 2008 to Sept. 2015. PIHs were defined as problematic in accordance with current consensus and dermatology guidelines to include cases with ulceration, bleeding, wound concerns, active or pending risk of functional deficits or obstruction (e.g., of airway or vision), visceral involvement, or future risk of permanent disfigurement [13]. All charts were examined for demographic detail, historical and clinical data, cardiac history and workup, and patient outcomes. Data were subsequently tabulated and analyzed. Descriptive statistics were used primarily as this study is aimed at detailing the clinically significant findings resulting from screening ECGs in this patient population.

### 2.1. Treatment Algorithm

All patients with PIH were screened at their first outpatient visit for perinatal complications, medical comorbidities, pulmonary and cardiovascular history, and family history of heart disease, cardiac arrhythmia, or unexplained sudden death. In all patients where propranolol therapy was recommended, a screening ECG was obtained based on institutional clinical protocol. A pediatric cardiologist interpreted all ECGs and made recommendations based upon results. In the setting of a normal ECG, patients were contacted by phone and instructed to pick up the prescription and begin propranolol. Instructions were provided verbally and described in a printed handout (written at a 5^th^-grade reading level) on how to administer the medication with care to give only during feeds and how to monitor for signs and symptoms of bradycardia, hypoglycemia, and hypotension. Caregivers were also instructed to follow up with their pediatrician within a week of starting propranolol for spot blood pressure and heart rate monitoring.

If the ECG suggested the presence of a structural or conduction defect, an echocardiogram was often obtained, a Cardiology referral was made, and propranolol therapy withheld until appropriate recommendations and monitoring was concluded by the cardiology service.

Propranolol was initiated as an outpatient treatment according to current consensus guidelines unless patient safety concerns dictated inpatient observation (e.g., age < 2 months or a significant known comorbidity such as chronic lung disease or PHACES). Initial propranolol dosing was typically 2 mg/kg/day divided three times daily. Patient caregivers were instructed to follow up with their pediatrician to measure heart rate and blood pressure. Patients were then seen 1 month after initiation of therapy and then at 3-month intervals. Weight-based adjustments to propranolol dosing were made throughout the treatment course at monthly intervals. Patients were typically treated until 1 year of age and then weaned from propranolol over 2-4 weeks at one-half the recommended dose. In patients with evidence of rebound growth or recurrent symptoms, treatment was restarted at 2 mg/kg/day divided three times daily and extended for at least 6 months with periodic dose adjustments for weight. This was most common in segmental, parotid, and airway lesions that have suspected prolonged proliferative and involution periods as previously reported in the literature [14].

## 3. Results

A total of 333 patients received propranolol for the treatment of PIH, 75% (*n* = 250) of whom were female. The average age at the time of starting therapy was 4.7 ± 4.3 months. Patients were treated for an average duration of 8.5 ± 5.0 months. The most common location of the lesion was the head and neck (*n* = 237) followed by the extremities (*n* = 24) and the trunk (*n* = 17). Fifty-five patients had hemangiomas at more than one site. Thirteen percent of patients (*n* = 44) were products of pregnancies complicated by hypertension/preeclampsia, placental abruption, or first trimester bleeding, and 19% (*n* = 64) were born prematurely defined as <38-week gestation. Thirteen patients had a history of twinning.

Forty-seven (14.8%) patients reported a total of 54 adverse effects after initiating propranolol. The majority of these (*n* = 31, 66%) were related to gastrointestinal complaints including exacerbation of reflux, decreased appetite, diarrhea, and constipation. There were 11 instances of persistent cough or wheezing. There were 8 instances of systemic symptoms including sleep disturbance and decreased energy. There were 5 instances of cardiac-related effects: 2 patients with symptomatic bradycardia, 2 with hypotension, and 1 whose family reported fainting. Nine patients stopped treatment early secondary to intolerance of the medication. There were at least 8 nonresponders to propranolol, and some of these also ended therapy early.

**Figure 1:**
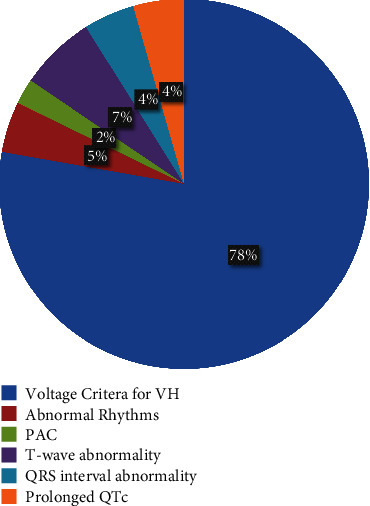
ECG abnormalities identified for patients within the cohort (*n* = 44).

ECG data were available in 317 patients (95%). The remainder had ECG performed prior to presentation, which was not available for review in the medical record. A total of 46 abnormal findings were present on *n* = 44 (13.9%) of study ECGs. The most common abnormal finding was “voltage criteria for ventricular hypertrophy” (*n* = 35). Two patients had abnormal rhythms: one first-degree atrioventricular (AV) block and one with occasional premature atrial contractions. Three patients had T wave abnormalities, 2 had an abnormal QRS interval, and 2 had a prolonged QTc (Figure 1). Of those patients with abnormal ECGs, 20.5% (9/44) had at least one follow-up visit with a pediatric cardiologist. That is, 2.9% of infants with PIH treated with propranolol required follow-up with a cardiologist. Despite these findings, no patient in the study was precluded from receiving propranolol by cardiology.

**Figure 2:**
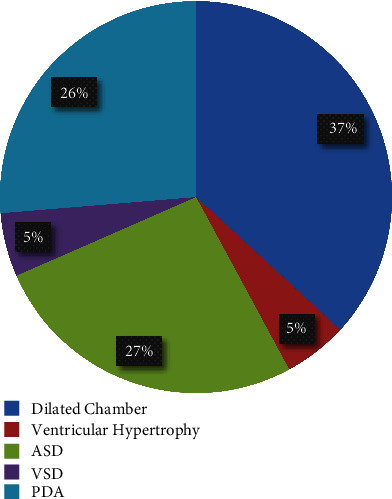
Echocardiogram abnormalities diagnosed (*n* = 20).

Echocardiography was ordered in the workup of PIH or in response to abnormal ECG findings in 31 patients. Echocardiogram results were also available in another 26 patients, which had coincidentally been ordered in the finding of a murmur on physical examination or in the workup of other medical comorbidity. Abnormal findings were present in 20 (35%) of studies. Overall, 20 out of 317 (0.6%) patients in the study had an echocardiographic abnormality. Seven patients had findings of at least one dilated chamber while one had ventricular hypertrophy. There were 5 atrial septal defects, 1 ventricular septal defect, and 5 patent ductus arteriosi (Figure 2). Five patients were found to have a valve abnormality. Thirty patients were also found to have a patent foramen ovale; however, given that the study was conducted on infants, this was not considered an abnormal finding.

Echocardiogram findings did not preclude the use of propranolol therapy in any affected infants as determined by the treating cardiologist.

## 4. Discussion

The discovery of the benefit of nonselective *β* blockade in the treatment of PIH represents a paradigm shift in treatment of these lesions. Additionally, the overall safety of this treatment, even in the context of some preexisting conditions, has made propranolol therapy the gold standard treatment modality for PIH [3]. Due to concern for side effects and relatively little experience in prescribing nonselective *β* blockers compared to cardiologists, some providers have been cautious in prescribing propranolol even when clinically indicated [15]. Though investigators have demonstrated that propranolol may cause significant changes in blood pressure [10] and ambulatory ECG findings [16], both studies showed that clinical symptoms did not occur. Jacks et al. investigated the usefulness of pretreatment Holter monitoring in 43 patients receiving propranolol for PIH [15]. There were no sustained arrhythmias, and the treatment plan was not changed in any of the patients. The data presented here reaffirm that propranolol is a safe therapy with a well-tolerated side effect profile. Adverse events were only reported in 47 (14%) patients. These were varied, and only nine (2.7%) patients had to cease propranolol therapy.

ECG in children is a reasonable screening test for underlying conduction system abnormalities; however, it is a relatively nonspecific screening test for structural heart disease. Only one patient (0.3%) in this cohort had underlying conduction system disease (first-degree AV block) and tolerated beta-blocker therapy without adverse event. While underlying structural heart disease may be clinically important to identify, essentially, no structural heart disease would be an absolute contraindication to beta-blocker therapy. In this study, no patient was precluded from receiving propranolol for PIH because of ECG or echocardiographic findings. Overall, only 2.9% of all patients required cardiology follow-up. Specifically, the findings of voltage criteria for ventricular hypertrophy, the most common abnormal finding on ECG, generated cardiology referrals in only 5 patients. All of these patients were found to have normal cardiovascular evaluation, received reassurance, and did not require any additional cardiology follow-up.

The number of patients needed to be screened before the diagnosis of congenital AV block is made is estimated to be 20,000 [17]. Though an ECG is a relatively inexpensive and extremely low morbidity investigation, its utility as a screening test in the study population was minimal. These results reinforce the idea that propranolol may be given for PIH without a screening ECG if there is no bradycardia, history of arrhythmia, family history of arrhythmia, or maternal connective tissue disease. Furthermore, many of the complications reported in the literature and thus contraindications to propranolol use pertain to noncardiac side effects such as hypoglycemia, especially in an infant with poor oral intake, bronchospasm, and sleep disorders [4, 5]. The finding of voltage criteria for ventricular hypertrophy may not require further investigation with an echocardiogram, and the initiation of therapy should not be excessively delayed.

Oral propranolol received FDA approval in 2014 and has since become the gold standard treatment for PIH. In 2019, the American Academy of Pediatrics released updated guidelines for the management of infantile hemangiomas [4]. This reaffirmed the strong recommendation for oral propranolol therapy in patients with PIH. In 2013, Drolet et al. proposed a more restrained use of ECG in pretreatment assessment recommending ECG only in patients with bradycardia, arrhythmia, or a family history of arrhythmia or congenital heart disease [8]. Though pretreatment ECG is briefly addressed in the newest AAP guidelines, the authors comment on the lack of data supporting the need for ECG but recommendations are still with ambiguity allowing for individualization of treatment based on patient factors. Our study offers further data showing that ECG does not regularly alter the clinical care of patients with PIH. As stated above, ECG only resulted in findings concerning enough to require follow-up with a pediatric cardiologist 2.9% of the time. No ECG findings amongst 317 patients precluded treatment with propranolol. Previous literature has reported a range of 6.5%-43% of all ECGs obtained prior to initiating propranolol therapy in PIH that were abnormal [10, 18–20]. Our study found that only 13.9% of patients had ECG abnormalities. The broad range reflects the need for continued investigation into this issue. To reiterate, though some have found limited clinical utility in screening ECG prior to initiation of propranolol therapy for PIH and proposed more restrained use of pretreatment ECG, AAP guidelines published in 2019 do not offer a definite algorithm for pretreatment testing. For this reason, this study is valuable as it offers further evidence that restrained use of ECG is appropriate. Additionally, this study further indicates the advantages and disadvantages of ECG prior to propranolol therapy. Early diagnosis of cardiac abnormalities through ECG/echocardiogram, in certain populations, is likely advantageous in the management of these patients. However, widespread use of ECG likely constitutes a vast expenditure that changes little in the management of PIH. This disadvantage is ever more applicable with the current push to reduce the mean cost of care.

By the nature of the protocol of our institution obtaining ECG on all patients starting propranolol outlined above, a limitation of our study became clear in that we were unable to stratify our patient's pre-ECG risk of having an abnormality. Though this limitation is notable, the findings are still germane to the discussion of pretreatment ECG and further bolster restrained use of pretreatment ECG. Other limitations of the study include the size of the population and the fact that it is single institution. Though size is limited, the authors could find no other study aimed primarily at ECG utility in this patient population with a cohort this large. A meta-analysis would be a valuable next step as there are now multiple studies assessing this issue. Further limitations exist in the retrospective nature of the investigation in being able to fully categorize all patient variables. The statistical weaknesses of this paper lie in the fact that it relies on descriptive statistics. Future studies would benefit from the development of a comparison group to allow for more robust statistical analysis.

## 5. Conclusion

Propranolol has become the gold standard treatment for PIH. Pretreatment ECG is commonly performed before initiation of propranolol, but data supporting this is lacking. This retrospective chart review sets out to assess the clinical utility of pretreatment ECG by assessing all patients at a single institution who underwent ECG prior to initiation of propranolol therapy. This showed that ECG abnormality is uncommon, and even when pretreatment ECG shows abnormality, propranolol is rarely, if ever, contraindicated. Patients rarely needed to be further evaluated by a cardiologist again showing the lack of utility in pretreatment ECG. This study supports the growing evidence showing that screening ECGs may be of limited use in the absence of concerning findings on initial history and physical examination.

## Data Availability

Data are available on request from the corresponding author, Gresham T. Richter (gtrichter@uams.edu).
